# BspK, a Serine Protease from the Predatory Bacterium Bdellovibrio bacteriovorus with Utility for Analysis of Therapeutic Antibodies

**DOI:** 10.1128/AEM.03037-16

**Published:** 2017-02-01

**Authors:** Eleni Bratanis, Henrik Molina, Andreas Naegeli, Mattias Collin, Rolf Lood

**Affiliations:** a Division of Infection Medicine, Department of Clinical Sciences Lund, Lund University, Lund, Sweden; b Proteomics Resource Center, The Rockefeller University, New York, New York, USA; McMaster University

**Keywords:** autoproteolysis, Bdellovibrio bacteriovorus, biotechnology, immunoglobulins, serine protease, therapeutic antibodies

## Abstract

The development of therapeutic and diagnostic antibodies is a rapidly growing field of research, being the fastest expanding group of products on the pharmaceutical market, and appropriate quality controls are crucial for their application. We have identified and characterized the serine protease termed BspK (Bdellovibrio serine protease K) from Bdellovibrio bacteriovorus and here show its activity on antibodies. Mutation of the serine residue at position 230 rendered the protease inactive. Further investigations of BspK enzymatic characteristics revealed autoproteolytic activity, resulting in numerous cleavage products. Two of the autoproteolytic cleavage sites in the BspK fusion protein were investigated in more detail and corresponded to cleavage after K_28_ and K_210_ in the N- and C-terminal parts of BspK, respectively. Further, BspK displayed stable enzymatic activity on IgG within the pH range of 6.0 to 9.5 and was inhibited in the presence of ZnCl_2_. BspK demonstrated preferential hydrolysis of human IgG1 compared to other immunoglobulins and isotypes, with hydrolysis of the heavy chain at position K_226_ generating two separate Fab fragments and an intact IgG Fc domain. Finally, we show that BspK preferentially cleaves its substrates C-terminally to lysines similar to the protease LysC. However, BspK displays a unique cleavage profile compared to several currently used proteases on the market.

**IMPORTANCE** The rapid development of novel therapeutic antibodies is partly hindered by difficulties in assessing their quality and safety. The lack of tools and methods facilitating such quality controls obstructs and delays the process of product approval, eventually affecting the patients in need of treatment. These difficulties in product evaluations indicate a need for new and comprehensive tools for such analysis. Additionally, recent concerns raised regarding the limitations of established products on the market (e.g., trypsin) further highlight a general need for a larger array of proteases with novel cleavage profiles to meet current and future needs, within both the life science industry and the academic research community.

## INTRODUCTION

The use of therapeutic antibodies in medical treatments and passive immunization dates back over a century; however, much has changed from the early serum therapies in the 1930s. Intravenous immunoglobulin (pooled immunoglobulin G [IgG] from 1,000 to 15,000 individuals per batch) is currently used extensively in the treatment of patients with antibody deficiencies (low dose) and as an immunomodulatory agent (high dose) in a wide range of immune and inflammatory disorders (1). However, issues including batch-to-batch variations and the low specificity of intravenous immunoglobulin have contributed to the development of therapeutic monoclonal antibodies (MAbs) (2). Human MAbs are, much owing to the general change toward biopharmaceuticals (biologics) in the pharmaceutical industry, well established in the treatment and diagnostics of disease. In 2014, there were over 30 MAb products approved for the market, and several hundred novel candidates are under clinical development (3). There are many advantages of protein therapeutics such as MAbs, compared to the classical chemical drugs, including high specificity for their targets, long half-lives, consistent pharmacokinetic properties, and low batch-to-batch variations (2). Advancements in molecular engineering technologies have in addition resulted in the minimization of the problem of immunogenicity (2, 3). Currently approved therapeutic MAbs are mainly of subclass IgG1 (4) and are used primarily in the treatment of various forms of cancer (5) and autoimmune diseases (2, 6). However, product approvals of biological agents, such as antibodies, that are naturally heterogeneous and undergo a wide range of posttranslational modifications (PTMs) require rigorous controls to guarantee their quality and safety prior to any clinical application. PTMs (e.g., glycosylation, methionine oxidation, deamination, glycation, and proteolytic processing) may affect protein structure, biological functionality, and stability, which may generate unwanted product variants. PTMs can be incorporated *in vivo* by natural cellular synthesis and processing or *in vitro* by manufacturing and processing (e.g., purification and storage), making quality controls of critical importance to ensure the efficacy and safety of these therapeutics (3, 7, 8). The current methodology used for such quality controls generally requires cleavage of the antibodies into smaller components in order to facilitate the analysis (3, 9). Peptide mapping is a commonly used strategy in such protein identification. In short, the method entails chemical or enzymatic cleavage of the protein into peptide fragments, followed by separation and identification of the fragments, usually performed by liquid chromatography-tandem mass spectrometry (LC-MS/MS). Proper, reliable, and reproducible sample preparation is critical for this analysis, and knowing the protein fragmentation profile is essential. The MS sensitivity is increased by fragmentation of the proteins into a wide range of overlapping peptides. Thus, to increase the peptide coverage, there is a need for a range of proteases with known cleavage profiles and overlapping hydrolytic activities (3, 9, 10). Additionally, antibody-binding and -modulating tools are greatly needed to facilitate basic research regarding antibody biology and functions. The recent expansion in the development of biological therapeutic agents such as MAbs has created considerable interest and demand for identifying novel immunoglobulin (Ig)-modulating enzymes. The concept of using bacterially derived proteins as biological agents to interact with IgG is already well established with the finding and application of proteins, including the IgG-binding proteins protein G and protein A (11, 12) and the IgG-specific hydrolases IdeS, EndoS, and EndoS2 (13–18).

Since the discovery of bacterial predation, much has been elucidated regarding the species diversity, distribution, and predatory strategies. Predatory bacteria are widely distributed, and recent findings indicate an intricate interplay between the predatory bacteria and their prey, with reciprocal effects acting as ecological balancers (19–21). Bacterial predation has also been proposed to play an important role in maintaining the biodiversity in the environment where they reside (20, 22). One of the most studied predatory bacteria is Bdellovibrio bacteriovorus, a small, Gram-negative, highly motile bacterium belonging to the class Deltaproteobacteria. B. bacteriovorus employs an endobiotic hunting strategy inferring a penetration of, and proliferation within, the prey (20). B. bacteriovorus invades the prey periplasm, where it resides in order to consume and utilize prey macromolecules as nutrients and biosynthetic materials, growing filamentously prior to septation into progeny cells. Following septation, the progeny is released by lysis of the host cell, beginning the cycle anew (23, 24).

Predatory bacteria have been investigated for their potential as live antibiotics during the last decades (25, 26). However, the use of live bacteria as therapeutics naturally raises questions and concerns regarding efficacy and safety. There have been several studies investigating the safety of B. bacteriovorus administration, and accumulating evidence supports the proposition that B. bacteriovorus is inherently nonpathogenic to mammals. Such evidence includes the discovery of B. bacteriovorus in the gastrointestinal tracts of mammals, showing a positive correlation between the prevalence of Bdellovibrio bacteria and the state of health (21, 27). The efficacy and safety of B. bacteriovorus administrations have also been investigated with human cells (28) and in several animal studies. The results of these studies show no signs of cytotoxicity or significant reduction in human cell viability and no adverse effects on animal growth or well-being (29, 30).

Predatory bacteria have recently gained renewed interest and are now being regarded as natural sources for the discovery of novel therapeutic agents and biotechnological tools due to their abundance of hydrolytic enzymes (31). Our interest in B. bacteriovorus is based on its vast array of hydrolytic proteins, having one of the highest numbers of protease genes per unit genome of all reported bacterial species to date (31). With this vast array of hydrolytic proteins, B. bacteriovorus has the potential to serve as a novel source of bacterially derived proteins, with applications within both basic research and the life science industry. This has been exemplified by the usage of whole B. bacteriovorus bacteria as a live lytic agent for the recovery of intracellular bioproducts (32). Here, we have isolated and characterized a novel serine protease from B. bacteriovorus with activity on immunoglobulins.

## RESULTS

### Identification of an IgG-modifying enzyme from B. bacteriovorus.

**FIG 1:**
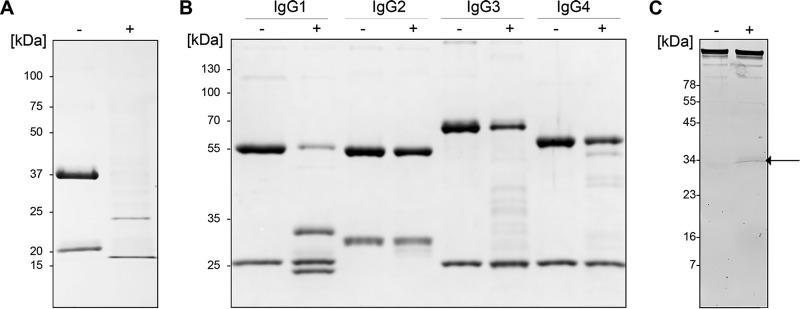
Bdellovibrio bacteriovorus hydrolyzes IgG. (A and B) Activity of crude B. bacteriovorus lysate against pooled hIgG (A) and against all hIgG subclasses (B). (C) Affinity purification of BspK from B. bacteriovorus crude lysate by the aprotinin-trastuzumab assay. An arrow indicates the affinity-purified band identified as Bd1962/BspK. Minus and plus signs indicate the absence and presence, respectively, of crude Bdellovibrio lysate in the sample.

An initial screening for IgG-modifying activities was performed with crude B. bacteriovorus lysate and revealed enzymatic activity on human IgG (hIgG), displaying a cleavage pattern suggestive of a single cleavage within the hinge region (Fig. 1A). Analysis of activity on the individual hIgG subclasses showed a preferential enzymatic activity on the heavy chain of hIgG1, resulting in two bands with molecular masses of ∼30 kDa and ∼23 kDa (under reducing conditions). Further analysis by nonreduced SDS-PAGE displayed two bands with molecular masses of ∼40 kDa and ∼50 kDa (data not shown), indicating hydrolysis above the disulfide bonds in the IgG hinge region, resulting in two separate Fab fragments and an intact IgG Fc domain, respectively. BspK had lower activity and several cleavage sites on hIgG3 and hIgG4. No activity was observed on hIgG2 (Fig. 1B).

In order to generate a partly purified material, proteins in the lysate were fractionated based on their biochemical properties, including solubility (ammonium sulfate precipitation), charge (ion-exchange chromatography), and size (ultrafiltration). The enzymatic activity was characterized by a systematic inhibition assay using a panel of protease inhibitors, followed by analysis with SDS-PAGE. The enzyme was inhibited by AEBSF [4-(2-aminoethyl)benzenesulfonyl fluoride hydrochloride], PMSF (phenylmethylsulfonyl fluoride), aprotinin, and EDTA (data not shown), which are general inhibitors of serine proteases and enzymes depending on divalent cations.

To identify the enzyme displaying the IgG-modifying activity of interest, we affinity purified the protein based on its interaction with aprotinin and hIgG1 (Fig. 1C). Aprotinin inhibits the enzymatic activity of the protease while still allowing it to bind to its substrate IgG1. Aprotinin-IgG1 complexes could then be purified on protein G Sepharose. Proteolytic activity and purity of the protease were verified by SDS-PAGE, and the resulting protein band was identified by mass spectrometry. The mass spectrometry analysis identified the protease as the 31.2-kDa Bdellovibrio bacteriovorus protein Bd1962, earlier annotated as a V8-like Glu-specific endopeptidase (NCBI GenBank) and as a trypsin-like (14% amino acid identity) serine protease, inferred by homology (UniProt). Due to the activity of Bd1962, we termed the protease BspK (Bdellovibrio serine protease K). BspK contains a conserved trypsin-like peptidase domain, comprising C_99_ to I_245_ (E value, 3.88e−17), as well as a likely signal peptide domain (amino acids 1 to 15). There are no additional conserved domains in the protein sequence. An XtalPred analysis of BspK revealed a predominantly helix-shaped N terminus, with the main part of the protein being structured in β-strands and a few regions of disorder (see Fig. S1 in the supplemental material).

### Expression and purification of recombinant BspK.

**FIG 2:**
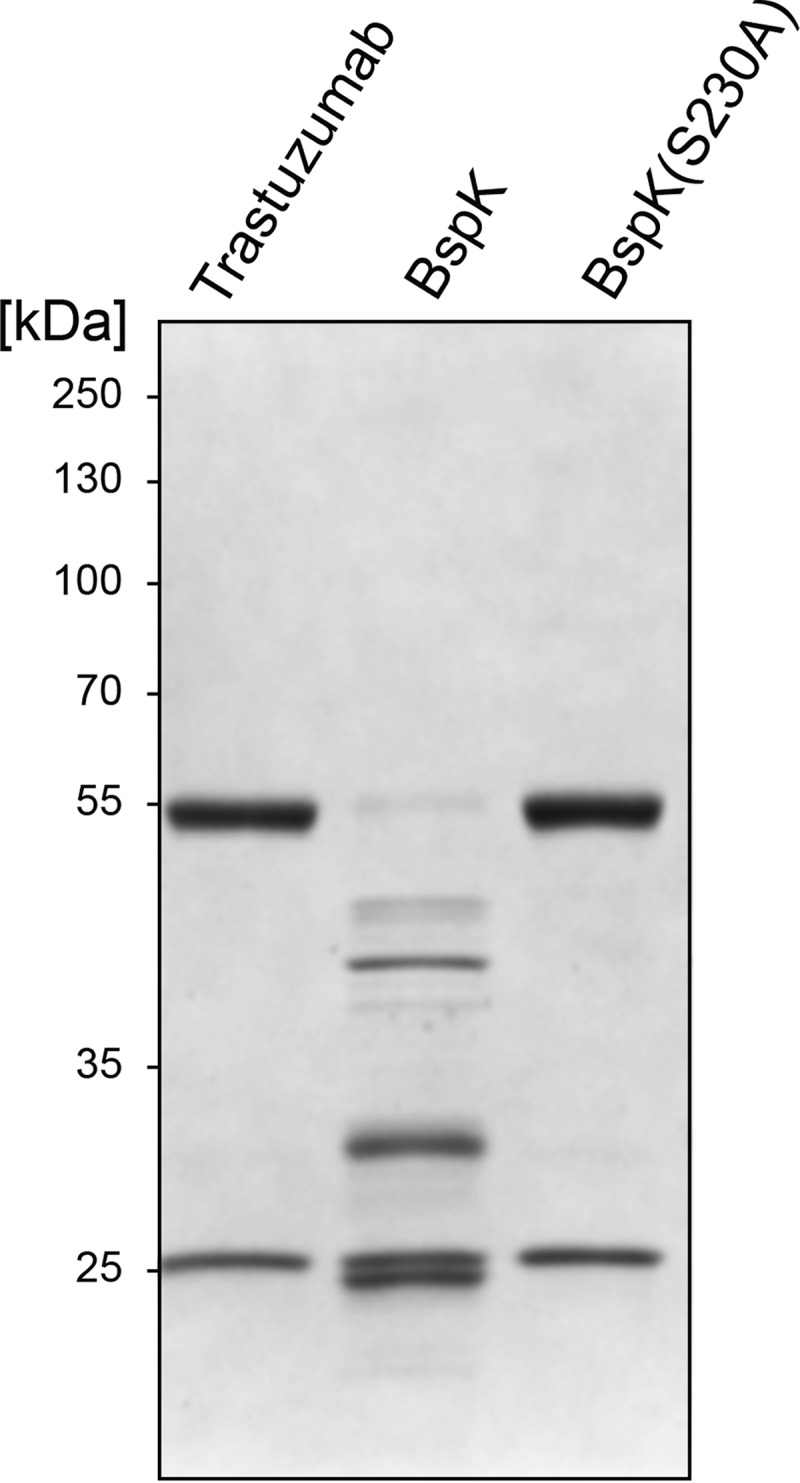
Serine 230 is required for BspK activity on trastuzumab. Trastuzumab was incubated with PBS, BspK, and BspK(S230A) for 2 h and separated by SDS-PAGE. Bands around ∼40 kDa represent autoproteolytic fragments of BspK.

Data generated by the protease inhibition assay established BspK as a serine protease. In order to generate an enzymatically inactive form of BspK, we performed site-directed mutagenesis of the serine in the catalytic triad. Recombinant BspK and its mutant BspK(S230A) were expressed using the IPTG (isopropyl-β-d-thiogalactopyranoside)-inducible pMAL-c5X-His vector. Following affinity purification on amylose resin, the enzymatic activity and purity of the proteins were analyzed by SDS-PAGE, verifying the activity of BspK and the inhibition of the IgG hydrolysis by an S230A mutation of BspK (Fig. 2). A protease inhibitor array for BspK activity confirmed the initial data on the crude lysate, verifying the activity as being mediated by a serine protease (Table 1).

**TABLE 1:** BspK activity is inhibited by serine protease inhibitors and EDTA^*a*^

Protease inhibitor	Inhibition of BspK activity
AEBSF	Yes
ALLN	No
Antipain	No
Aprotinin	Yes
Bestatin	No
Chymostatin	No
E64	No
EDTA	Yes
Leupeptin	No
Pepstatin	No
Phosphoramidon	No
PMSF	Yes

a The ability of BspK to hydrolyze IgG was completely inhibited or was unaffected in the presence of specific protease inhibitors. ALLN, *N*-acetyl-l-leucyl-l-leucyl-l-norleucinal.

### Biochemical characteristics of BspK.

**FIG 3:**
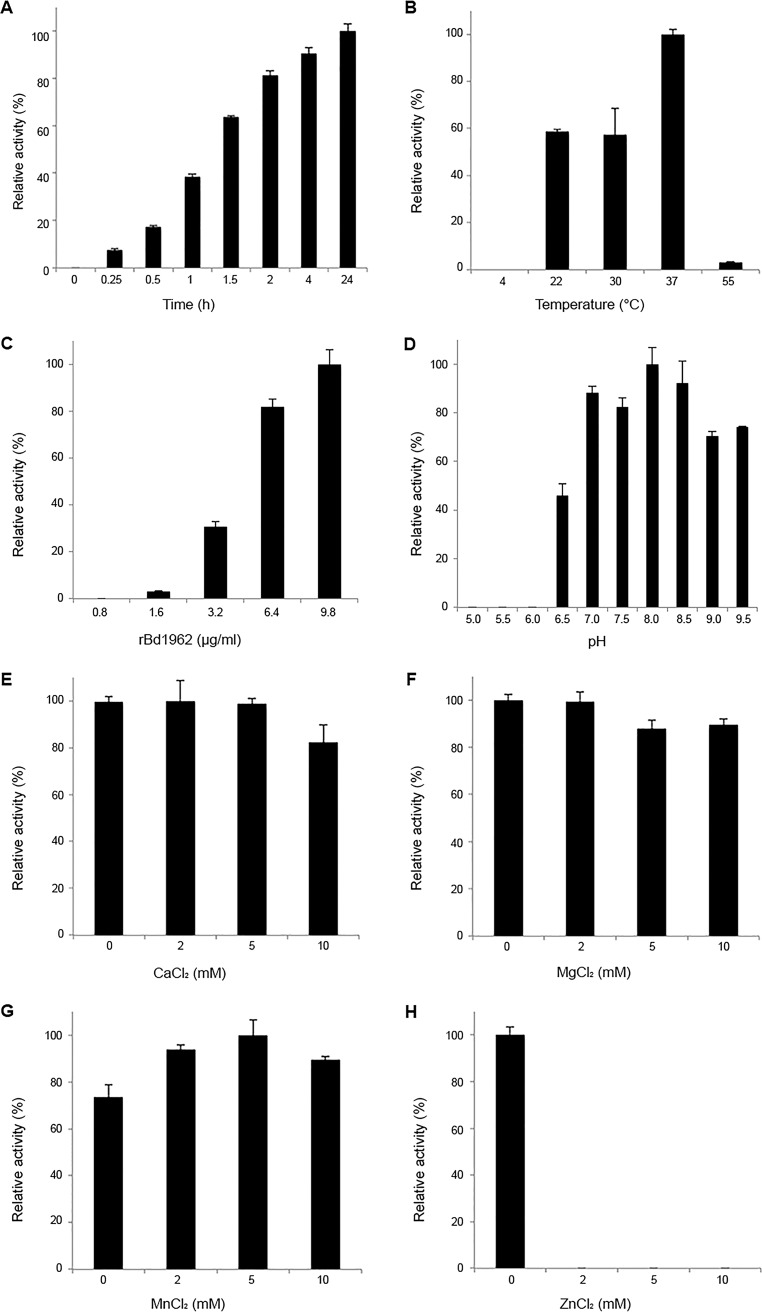
Enzymatic characteristics and optimal conditions for BspK hydrolysis of trastuzumab. (A) BspK enzymatic activity over time; (B) BspK activity with increasing incubation temperatures (4 to 55°C); (C) dose response assay of BspK; (D) investigation of pH optimum (5.0 to 9.5) for BspK activity; (E to H) BspK activity in the presence of increasing concentrations of divalent cations (0 to 10 mM). The standard concentration of BspK used for the activity assays was 3.2 μg/ml. Unless otherwise stated, all experiments were conducted with overnight incubations at 37°C in 20 mM Tris buffer, pH 6.8, with 2.7 mM CaCl_2_. The results are presented as relative activities with respect to the maximal activity within the individual assay.

To minimize the variability between assays, we used trastuzumab, a human therapeutic monoclonal IgG1 antibody, as the substrate. Investigation of the optimal biochemical conditions for BspK activity on trastuzumab showed that the proteolytic activity has a pH optimum around pH 7 to 8, with activity between pH 6.5 and 9.5 (Fig. 3D). Increasing NaCl concentrations, ranging from 50 mM to 1 M, did not significantly affect the enzymatic activity of BspK (data not shown).

**FIG 4:**
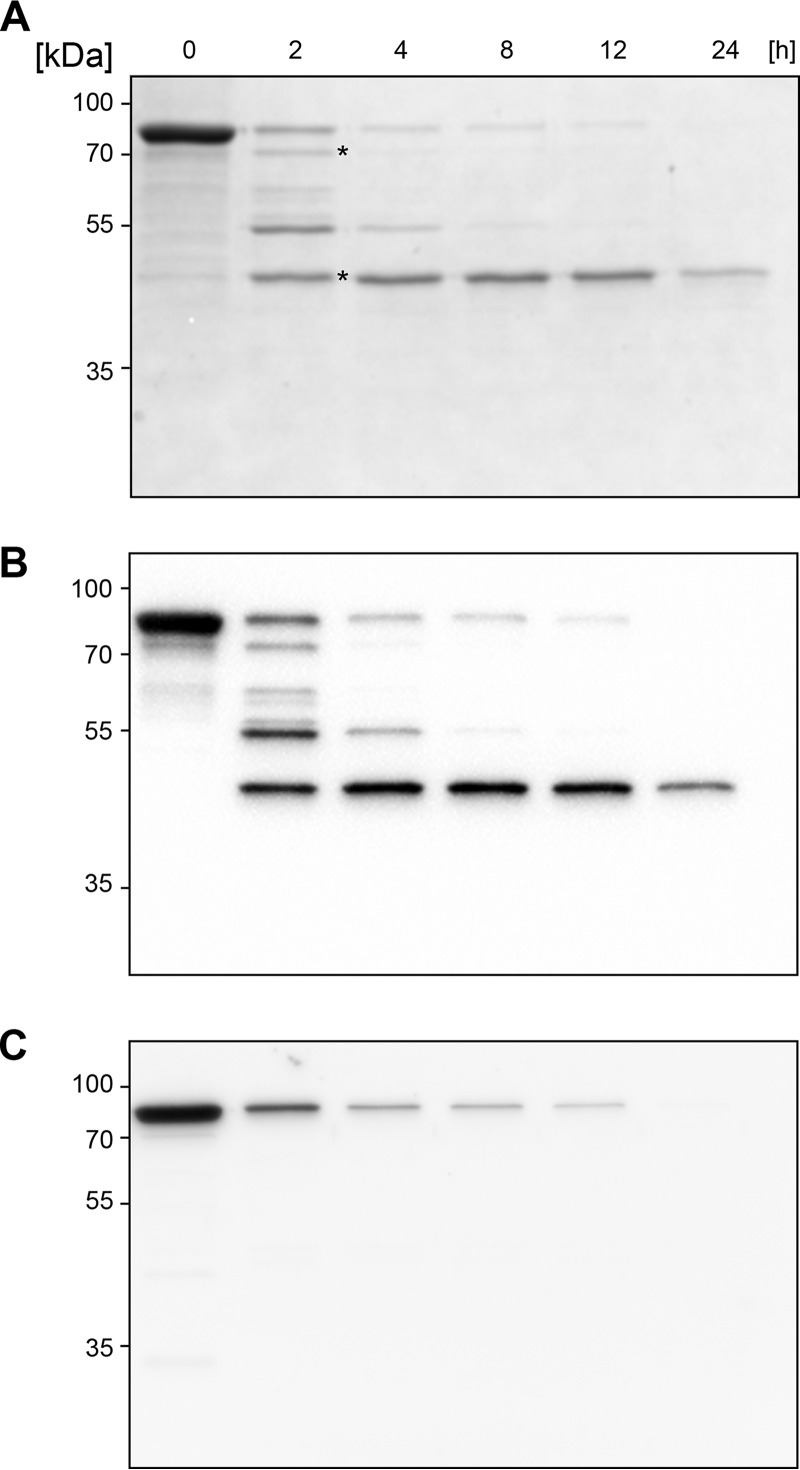
BspK displays autoproteolytic activity. (A) SDS-PAGE showing BspK autoproteolysis; (B) anti-MBP antibody Western blot; (C) anti-His antibody Western blot. Intact recombinant BspK (MBP-BspK-His) has a molecular mass of ∼74 kDa; MBP has a molecular mass of 42.5 kDa. Bands marked with an asterisk indicate bands for which the cleavage site was determined.

The activity of BspK on trastuzumab peaked at 37°C but displayed activity even at lower temperatures (22 to 30°C). No activity could be detected at lower (4°C) or higher (55°C) temperatures (Fig. 3B). The activity was dose dependent (Fig. 3C) and could be visualized already after 15 min of incubation, with increasing activity up to 24 h (Fig. 3A). The stability of BspK (autoproteolysis) correlates with the enzymatic activity shown on trastuzumab (Fig. S2). A small discrepancy with regard to the ability of the different antibodies (maltose-binding protein [MBP] and MBP-BspK-His antibodies) to bind to their target is likely to be related to the usage of distinct antibodies (Fig. S2; Fig. 4).

Our previous results demonstrated an inhibitory effect of EDTA on BspK activity, which implies a dependence of divalent cations. To further investigate this phenomenon, we studied what effects increasing concentrations (0 to 10 mM) of CaCl_2_, MgCl_2_, MnCl_2_, and ZnCl_2_ would have on the enzymatic activity of BspK. The addition of CaCl_2_, MgCl_2_, and MnCl_2_ had no significant effect on the activity, due to an already high activity at 0 mM. Interestingly, the presence of ZnCl_2_ resulted in a complete loss of activity (Fig. 3E to H).

### BspK displays autoproteolytic activity.

**FIG 5:**
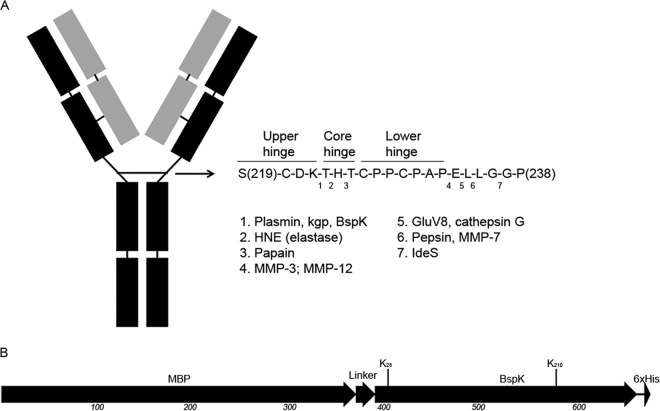
Schematic IgG1 and BspK fusion protein cleavage maps. Schematic figures of the BspK cleavage site in the IgG1 hinge region compared to other known enzymes with hydrolytic activity on IgG1 (A) and the BspK fusion protein showing the N-terminal MBP and the C-terminal His tag (B). Vertical lines indicate the BspK autoproteolytic cleavage sites at K_28_ and K_210_.

During our initial studies, we noted that BspK displayed autoproteolytic activity, later defined as C-terminal processing of the fusion protein (Fig. 4). It is important to note that the intact BspK fusion protein migrates somewhat higher on an SDS-PAGE gel than its expected theoretical mass (e.g., 74.3 kDa) (Fig. 4A). Two of the cleavage products, represented by bands at ∼45 kDa and ∼70 kDa (Fig. 4A), resulting from BspK autoproteolytic hydrolysis, were further characterized by mass spectrometry in regard to their cleavage sites. The cleavage sites corresponded to cleavage after K_28_ and K_210_ in the fusion protein (Fig. 5B). The K_28_ and K_210_ cleavage sites correspond well to the observed masses of the fragments, i.e., 44.34 kDa (e.g., the MBP domain and a small part of the BspK domain upstream of K_28_) and 64.11 kDa (e.g., the main part of the fusion protein, lacking only the C-terminal part from K_210_), respectively, taking into account the partly altered migration of BspK in polyacrylamide gels (Fig. 4A). It should be noted that during assessment of the cleavage sites, other less frequent cleavage sites could also be detected, indicating a presence of multiple different cleavage products in the processed gel bands. This is in accordance with our data, demonstrating that BspK undergoes further hydrolysis, finally rendering most cleavage products undetectable by SDS-PAGE and Western blotting (Fig. 4). Thus, the presence of a minor subpopulation of protein with a diverse cleavage pattern in the gel band is only to be expected. Our results also show that the native BspK displays autoproteolysis, corresponding to the observed pattern for the recombinant protease (Fig. S3). The shift of the lower band correlates well with the predicted size of the identified autoproteolytic cleavage product of BspK following cleavage at K_210_ (Fig. 5B).

### Activity of BspK autoproteolytic fragments.

The confirmation and characterization of the autoproteolytic activity of BspK led to further investigations regarding the enzymatic activity of the autoproteolytic products. The results following a preincubation of BspK, allowing the processing of the full-length MBP-BspK-His protein into the autoproteolytic intermediate BspK products (Fig. 4 and 5B), prior to incubation with IgG1 suggests that certain autoproteolytic BspK products retain enzymatic activity. The results also show that BspK cleavage products display higher enzymatic activity than BspK kept at −80°C (Fig. S4), indicating that the processed BspK is more enzymatically active than intact MBP-BspK-His protease.

### BspK hydrolyzes immunoglobulins.

**FIG 6:**
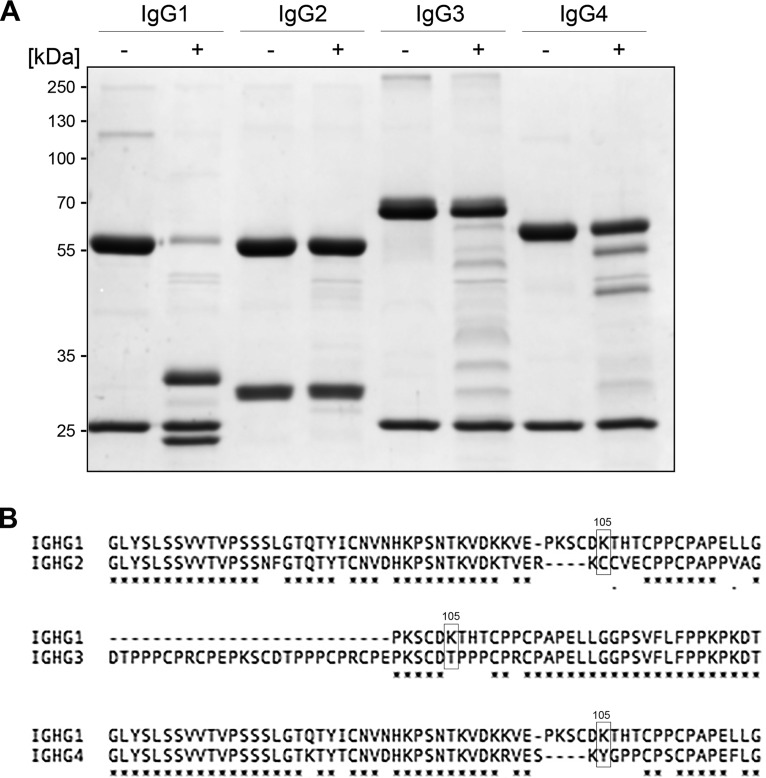
BspK hydrolyzes predominantly human IgG1. (A) BspK was incubated with human IgG subclasses (1 to 4) for 2 h and analyzed on an SDS-PAGE gel. Minus and plus signs denote the absence and presence of BspK in the sample, respectively. (B) Sequence alignment of human IgG constant regions displaying the similarities and variations between the hinge regions of IgG1 and IgG2 to IgG4. Marked amino acid residues at position 105 represent the BspK cleavage sites (the identified BspK cleavage site at K_226_ in trastuzumab corresponds to K_105_ in the IGHG1 sequence used in the sequence alignment).

In order to investigate the substrate specificity of BspK, we incubated BspK with several immunoglobulins. BspK demonstrated activity against several different immunoglobulins after 2 h and a broader spectrum of activity after 24 h (Table 2). Further, BspK had a preferential activity on native human IgG1 cleaving specifically after K_226_ in the IgG1 hinge region, generating two separate Fab fragments and an intact IgG Fc domain (Fig. 5 and 6A). The sequence alignment of the IgG1 to IgG4 hinge regions (Fig. 6B) displays sequence variations in the region comprising the BspK cleavage site in IgG1. The identified BspK cleavage site in trastuzumab (K_226_) corresponds to K_105_ in the IgG1 sequence used in the alignment, and as shown, K_105_ is exclusive to the IgG1 hinge.

**TABLE 2:** BspK hydrolysis of immunoglobulins after 2 and 24 h^*a*^

Immunoglobulin	Activity after:	
	2 h	24 h
Human IgG1	+	+
Human IgG2	−	−
Human IgG3	−	+
Human IgG4	−	+
Human IgD	+	+
Human IgE	+	+
Human IgA	−	+
Human secretory IgA	+	+
Human IgM	−	+
Mouse IgG1	−	−
Mouse IgG2b	+	+
Mouse IgG3	−	−
Rabbit IgG	+	+
Goat IgG	−	+
Bovine IgG	−	+
Guinea pig IgG	−	+
Pig IgG	−	−
Sheep IgG	−	+
Horse IgG	+	+
Donkey IgG	−	−
Dog IgG	−	+

a BspK was incubated with immunoglobulins for 2 to 24 h, and activity was analyzed by SDS-PAGE.

### Identification of BspK preferential sites of hydrolytic activity.

Incubation of BspK with gelatin or casein resulted in no measurable hydrolytic activity (data not shown). However, further screening with several short peptides revealed the ability of BspK to generate fragments corresponding to hydrolysis C-terminal to lysines, generating a similar cleavage pattern as the Lysobacter enzymogenes serine protease LysC (33) (Fig. S5). Peptides lacking lysines, or with C-terminal lysines only, were unaffected by BspK (Table 3). Hydrolysis of apo-myoglobulin by BspK resulted in a unique cleavage pattern in comparison to trypsin and LysC (Fig. S6). Of note, in contrast to trypsin and LysC, BspK displayed no significant limitation in hydrolyzing K-P bonds, as demonstrated by the degradation of the peptide GHHEAELKPLAQSHATK to GHHEAELK and PLAQSHATK (Fig. S6).

## DISCUSSION

The general shift in the pharmaceutical industry toward biologics is driving the rapid development of biopharmaceutical agents, including the development of MAbs. The progress toward the use of complex biological molecules as therapeutic agents has created a need for novel tools and innovative methods to ensure their quality and safety (3). The use of bacterial enzymes as biotechnological tools is already well established, and we propose that B. bacteriovorus, with its vast array of hydrolytic enzymes, is an excellent, as yet underexploited source to identify novel biotechnological tools.

A crude lysate from B. bacteriovorus displayed potent activity against human IgG. Taking advantage of the specific interaction of the protease with aprotinin and hIgG1, we were able to affinity purify the IgG protease and identify it as Bd1962, later denoted BspK. A prediction with SignalP 3.0 strongly indicated (0.995 probability) that BspK contains a signal peptide, suggesting that it is a secreted protein. This is further supported by previous findings showing that BspK (previously Bd1962) is secreted by host-independent (HI) B. bacteriovorus (34, 35). Taken together, these findings strongly suggest that BspK is a secreted protein, conserved in both host-dependent (HD) and HI B. bacteriovorus. Several studies of gene regulation during the different phases of the B. bacteriovorus life cycle have reported variations in the gene expression of *bspK* in the attack phase versus the intraperiplasmic growth phase. Lambert et al. identified *bspK* as being downregulated once B. bacteriovorus enters a bdelloplast state (e.g., during the initial infection of the prey) (36). However, Karunker et al. demonstrated a significant increase in gene expression of *bspK* when B. bacteriovorus changed from its attack phase (free-swimming phase) to its growth phase (37). The two studies are, however, not necessarily contradictory, since Lambert et al. studied the gene expression during bdelloplast formation (early event in the infection) and Karunker et al. did so at a later stage of infection, where it may be assumed that several enzymes involved in prey predation are expressed.

A comparative genetic analysis (BLASTn) revealed that the *bspK* gene is conserved among, and is specific for, B. bacteriovorus strains HD100, 109J, and Tiberius. On the protein level, a multiple-sequence alignment (MSA) with BspK and various bacterial proteases showed that BspK has some similarity to trypsin from Omnitrophica bacterium but is quite distant from other currently known proteases (Fig. S7). A protein BLAST on BspK limited to the Bdellovibrionales order, followed by a ClustalW alignment, resulted in a comprehensive guide tree of different predatory bacteria with homologues to BspK (Fig. S8). Of interest, B. bacteriovorus has a second gene (*bd0994*) in its genome with high similarity to *bspK*. Thus, the presence of BspK, or highly similar proteins, seems to be conserved among predatory bacteria. Further characterization of the proteolytic specificity revealed a preference for hydrolysis C-terminal to lysines similar to the activity of the L. enzymogenes serine protease LysC (33).

Another widely used protease is trypsin, which due to its detailed characterization, robustness, and well-documented specificity has become the enzyme of choice for the majority of high-throughput proteomics experiments. Trypsin hydrolyzes its substrates C-terminally to lysines (Lys) and arginines (Arg), and it is well recognized that this activity is reduced considerably at sites where Lys or Arg precede proline (Pro) in the substrate sequence (9, 38). Our analysis of BspK specificity did not indicate any such restrictions of BspK hydrolysis (Fig. S6).

Our results revealed a specific cleavage of trastuzumab by BspK after the lysine residue at position 226, resulting in the cleavage of IgG into two separate Fab fragments and an intact Fc fragment, resembling the previously described activity of the Porphyromonas gingivalis cysteine protease Kgp (39). We also observed a general activity by BspK on a spectrum of immunoglobulin isotypes and subclasses, from several species. A correlation between prolonged incubation and an increasing substrate activity indicated that BspK displays hydrolysis on a broader spectrum of substrates after prolonged incubations. Interestingly, no further hydrolysis of trastuzumab was observed under any of the tested conditions. As the majority of all therapeutic antibodies, including trastuzumab, are of subclass IgG1 (4), the specificity of BspK makes it suitable as a tool for preparation of therapeutic antibodies prior to mass spectrometry analysis.

There are most likely several reasons for the specific activity of BspK on IgG1 compared to the other IgG subclasses, including site specificity of the protease and the accessibility of this site in the IgG1 hinge region. The IgG subclasses are generally highly homologous; however, there are variations, found mainly in the hinge region and the N-terminal CH2 domain (40). These sequence diversities result in substantial structural variations between the hinge regions of the different subclasses, affecting the flexibility and ultimately the antibody conformation (40). Our characterization of BspK activity shows preferential hydrolysis C-terminal to lysines and specific hydrolysis at K_226_ in trastuzumab (Fig. 6A). A sequence alignment of the IgG subclasses (Fig. 6B) shows that the identified site of BspK hydrolysis is specific to the IgG1 hinge region.

Based on the identification of BspK with aprotinin and hIgG1, we also hypothesize that BspK might have separate enzymatic and substrate-binding domains. This may imply that BspK hydrolysis requires specific substrate binding (structural or motif specific) for hydrolytic activity, possibly explaining the inability of BspK to hydrolyze casein proteins, which contain many Lys-Xaa bonds. Further research is needed to reveal the details of the BspK structure.

The Ig domain, primarily recognized for its role in the immune system, has been shown to be widespread in nature, found in a vast array of proteins with diverse biological functions. The Ig-like fold comprising the three-dimensional structure of the Ig-like domain has been identified in various species, including both eukaryotes and prokaryotes (41, 42). In bacteria, the Ig-like fold can be found in proteins such as adhesins, chaperones, transporters, and enzymes, among others. Interestingly, the Ig-like domain is frequently found and is widely distributed in Escherichia coli and other enterobacteria (42), bacteria known to colonize the intestinal tracts of mammalians. Together with aquatic and terrestrial environments, the gastrointestinal tract is also where B. bacteriovorus is found (31). Consequently, B. bacteriovorus is unlikely to encounter IgG in any of its natural environments, as IgG is found mainly in the circulation. However, as a predator within the gastrointestinal tract, an organ known for its vast microbiota, B. bacteriovorus is certain to encounter bacteria carrying proteins with Ig-like folds. In contrast, the number of proteins predicted to have an Ig fold in B. bacteriovorus is limited (*n* = 1; Superfamily v1.75), if even present (*n* = 0; Pfam v30.0) and should thus not be prone to degradation by BspK. Thus, we hypothesize that BspK hydrolysis requires specific substrate binding for hydrolytic activity and that this substrate possibly is a motif or structure found in the Ig-like domain. By this reasoning, we suggest that the specific activity on IgG1 most likely is coincidental, based on a resemblance with the true BspK substrate(s), and that BspK most likely has evolved to hydrolyze other bacterial proteins containing an Ig-like fold.

The addition of EDTA completely inhibited the activity of BspK, indicating its dependence on divalent cations for activity. However, when the dependence on specific divalent cations was analyzed, no addition of ions was necessary for activity, indicating that trace amounts of ions in the reaction buffer were enough to support full activity (Fig. 3). In support of previous data, rebuffering of BspK with ultrapure water (Milli-Q H_2_O) rendered it virtually inactive (data not shown). Interestingly, the complete inhibition of BspK by the addition of ZnCl_2_ suggests that there are specific requirements to the nature of the ions, resulting in either enzymatic activity or inhibition.

The lifestyle of B. bacteriovorus requires it to produce a vast array of hydrolytic enzymes, many of which have not been identified or characterized in detail. This assortment of hydrolases likely comprises a variety of both substrate-specific and broadly acting enzymes. Our results show that the autoproteolysis of BspK results in enzymatically active cleavage products. These results also indicate that the BspK fragments have a higher enzymatic activity than the intact MBP-BspK-His fusion protein. Enzymes are often produced as inactive precursors (e.g., zymogens) that require a biochemical change, such as hydrolysis, to become an active enzyme. The production of BspK as a zymogen might explain the increased activity of BspK following initial autoproteolysis. However, separately aligning the BspK homologues within the BALO_8 group revealed that K_28_ is conserved in 75% and K_210_ in only 25% of the strains (Fig. S9). Whether the BspK homologues from these other groups have different autoproteolytic behavior remains to be elucidated, but our data strongly suggest that these regions are highly variable. Furthermore, the results show that a prolonged incubation of BspK leads to a complete degradation of the protease, suggesting that the autoproteolytic activity of BspK could serve as a self-regulating mechanism, protecting against excessive and/or unwanted hydrolysis. Autoproteolysis of proteases is common, and both trypsin and Kgp display similar self-degrading properties (43). However, in contrast to that hypothesis, we detect an increased hydrolytic activity in BspK samples that had undergone significant autoproteolysis (Fig. S4). Thus, even though it may be possible to stabilize BspK through protein engineering (e.g., replacement of exposed lysines), that may be detrimental to the enzymatic activity. Further experiments are needed to clarify this.

Regarding the potential applications of B. bacteriovorus as a live antibiotic, the effects of BspK *in vivo* are unknown. Hydrolysis of IgG, a major component of the humoral immune system, by BspK *in vivo* may have serious negative implications for the immune system. However, none of the *in vivo* studies (27, 29, 30) investigating the effect of B. bacteriovorus administration indicate any such side effects. Furthermore, the aspect of localization cannot be ignored when discussing BspK hydrolysis of IgG *in vivo*. IgG antibodies are found mainly in the circulation, while B. bacteriovorus resides in the gastrointestinal tract, implying that the enzyme and its substrate do not naturally come in contact. Based on the autoproteolytic property of BspK, any leakage into the circulation, where it could hydrolyze the antibodies, seems highly unlikely.

Finally, the limitation of trypsin activity and the recently raised concerns regarding the general acceptance of inaccurate rules of trypsin specificity (e.g., ability to hydrolyze R/K-P bonds and a secondary low degree of proteolysis at asparagine residues) (9, 43) are good examples stressing the need for novel biotechnological tools and methods, as well as the importance of knowing the precise cleavage profile of the applied protease. The rapid development of biologics and the increasing application of mass spectrometry-based proteomics methods, requiring cleavage of intact proteins into smaller peptides (9), have emphasized a need for a larger array of novel biotechnological tools. The current and future needs, within both the life science industry and the academic research community, would best be met by a spectrum of hydrolytic enzymes displaying unique and complementary cleavage profiles. We propose that B. bacteriovorus might serve as a source for novel biotechnological tools, demonstrated by the identification and characterization of BspK, a putative new tool in such a toolbox, displaying a novel cleavage profile compared to those of previously characterized proteases currently available on the market.

## MATERIALS AND METHODS

### Bacterial strains and growth condition.

B. bacteriovorus HD100 was propagated and routinely cultured with E. coli as prey, as previously described by Lambert et al. (23). Lysis was determined by visual clearing of the culture medium in comparison to a noninfected E. coli control. E. coli strains TOP10 and BL21(DE3)/pLysE were used for cloning, preparation of plasmids, and expression of recombinant proteins. E. coli was cultured in LB medium supplemented with 100 μg/ml ampicillin, 34 μg/ml chloramphenicol, and 1% glucose where appropriate.

### Purification of BspK from Bdellovibrio lysate.

A sterile filtered (0.22-μm pore size) B. bacteriovorus-E. coli (predator-prey) overnight culture was precipitated by a two-step ammonium sulfate precipitation (50 to 80%). Precipitated proteins were resolubilized in 20 mM Tris, pH 6.8, followed by ion-exchange chromatography (HiTrap Q FF, pH 6.8) (GE Healthcare Biosciences AB, Uppsala, Sweden) and ultrafiltration (Amicon Ultracel-50) (Merck Millipore Ltd., Tullagreen, Carrigtwohill, Ireland). The flowthrough was concentrated and rebuffered to a Tris buffer (20 mM, pH 6.8) using Ultracel-10 centrifugal filters (Merck Millipore). Proteolytic activity and purity were verified by SDS-PAGE.

### Affinity purification of BspK.

BspK purified from Bdellovibrio lysate was preincubated with aprotinin (Thermo Scientific, Rockford, IL, USA), after which hIgG1 (trastuzumab) was added to the reaction mixture. The sample was applied to a protein G Sepharose column (Ab spin trap; GE Healthcare) according to the manufacturer's instructions. Eluted proteins were separated on a Tricine SDS-PAGE gel (Novex 16%; Thermo Scientific), and protein bands were identified using mass spectrometry.

Trastuzumab is a humanized MAb originally developed and used for the treatment of HER2-overexpressing metastatic breast cancer (44). Trastuzumab was used as the substrate of choice due to the preferential activity of BspK on IgG1, its availability, and its monoclonal property, minimizing the substrate variability between experiments.

### In-gel digestion and mass spectrometry.

A band corresponding to the predicted size of Bd1962/BspK was excised from the Tricine gel following aprotinin-hIgG1 affinity purification. The gel was sectioned into smaller pieces, followed by destaining, washing, and dehydration with acetonitrile (ACN; 50%). Reduction of disulfide bonds with tris(2-carboxyethyl)phosphine (TCEP) solution (10 mM in 100 mM ammonium bicarbonate, pH 8.0) and alkylation of cysteines with iodoacetamide (IAA; 15 mM in 100 mM ammonium bicarbonate, pH 8.0) were followed by trypsin (Promega) digestion and finally peptide extraction with formic acid (FA) and ACN (50%). Peptides were analyzed by LC-MS/MS using an Easy-nLC 1000 system equipped with an EasySpray ES802 column connected to a Q-Exactive plus mass spectrometer with an EasySpray electrospray ion source (all from Thermo Scientific). MS/MS spectra were acquired in a data-dependent (DDA) mode, with selection for the 15 most abundant precursor ions (400 to 1,600 *m/z*, 70,000 resolving power at 200 *m/z*) for fragmentation (17,500 resolving power at 200 *m/z*) each cycle. The raw data were searched using the Trans Proteomic Pipeline 4.7 (45) against a decoyed sequence database of the B. bacteriovorus HD100 proteome retrieved from the UniProt database (ID UP000008080). The false discovery rate was set to 1%.

### Cloning and site-directed mutagenesis of *bspK* from Bdellovibrio.

*bspK* lacking the region encoding the N-terminal signal peptide, as determined using SignalP 3.0 (46), was amplified by PCR using primers 5′-TATCATATGGCAACGTTCTCAGTCGG-3′ and 5′ ATAGGATCCCTGTTTCAAAGCCTGAGA-3′ with Phusion Hot Start II high-fidelity polymerase (Fisher Scientific). (Underlined sequences indicate added restriction sites [NdeI and BamHI, respectively].) A catalytic triad serine mutant (S230A) was introduced by using the Q5 site-directed mutagenesis kit (NEB, Ipswich, MA) with primers 5′-TGGTGGCAACGCTGGTTCTGCTG-3′ and 5′-TAAGTGTCCAGATTAGTTACGAAGTAGC-3′, according to the manufacturer's instructions. The amplified products were cloned into the pMAL-c5X-His vector (NEB), propagated in E. coli TOP10 cells (Invitrogen), and subsequently transformed into E. coli BL21(DE3)/pLysE cells (Invitrogen) for recombinant protein expression.

### Recombinant expression and purification of BspK.

BspK and its variant BspK(S230A) were produced in E. coli BL21(DE3)/pLysE cells as MBP fusion proteins. Bacteria were cultured (37°C, 225 rpm) until the optical density at 620 nm (OD_620_) reached 0.4 when recombinant protein expression was induced with 0.1 mM IPTG. Protein expression was continued for 3 h (20°C, 225 rpm), after which cells were collected and lysed with BugBuster (EMD Millipore Corp., Billerica, MA, USA) supplemented with benzonase (EMD Millipore Corp.). Soluble proteins were affinity purified on amylose resin (NEB) according to the manufacturer's instructions. Finally, the recombinant protein was purified by ion-exchange chromatography (HiTrap Q FF, pH 7.8) (GE Healthcare). The sample was rebuffered with Amicon filters to 20 mM Tris-HCl, pH 6.8.

### Properties and optimal conditions of BspK enzymatic activity.

The optimal biochemical condition for BspK enzymatic activity was evaluated by altering specific parameters in a standardized activity assay (unless otherwise stated, all experiments were conducted with overnight incubations at 37°C in 20 mM Tris buffer, pH 6.8, with 2.7 mM CaCl_2_). pH dependence was addressed by using buffers with overlapping pH ranges (sodium acetate buffer, pH 5.0 to 5.5; Tris buffer, pH 6.0 to 9.5). The effects of NaCl (50 mM to 1 M), temperature (4°C to 55°C), and incubation time (0 min to 24 h) on BspK proteolytic activity were also determined. Characterization of the proteolytic activity was investigated with a panel of protease inhibitors (G-Biosciences, Geno Technology Inc., USA) according to the manufacturer's instructions. Trastuzumab was used as the substrate to determine BspK activity. The hydrolysis was densitometrically quantified using Image Lab software (version 5.2.1; Bio-Rad Laboratories). The results are given as relative activities with respect to the maximal activity within the individual assay. The autoproteolytic activity of BspK was also evaluated in these assays.

### Autoproteolytic activity of recombinant BspK.

BspK and BspK(S230A) were incubated at 37°C for a set amount of time and separated by SDS-PAGE under reducing conditions. Proteins were transferred to polyvinylidene difluoride (PVDF) membranes using Trans-Blot Turbo equipment (Bio-Rad Laboratories). Membranes were blocked with 5% (wt/vol) blotting-grade blocker (Bio-Rad Laboratories) in PBST (phosphate-buffered saline with Tween 20). Antibodies directed to MBP (anti-MBP monoclonal, mouse antibody, HRP conjugated; NEB) or the His tag (anti-6×His tag, mouse antibody; Abcam) and Alexa Fluor 568-conjugated donkey anti-mouse IgG antibody (Thermo Scientific) were used to visualize the fusion proteins in a ChemiDoc MP Imager (Bio-Rad, USA).

### Autoproteolytic activity of native BspK.

A sterile filtered (0.22-μm pore size) B. bacteriovorus-E. coli (predator-prey) overnight culture was concentrated 100-fold for subsequent analysis by Western blotting. Proteins were separated by SDS-PAGE under reducing conditions, followed by transfer to PVDF membranes using Trans-Blot Turbo equipment (Bio-Rad Laboratories). Membranes were blocked with 5% (wt/vol) blotting-grade blocker (Bio-Rad Laboratories) in PBST. Antibodies directed to BspK (anti-BspK/anti-MBP polyclonal, rabbit antibody) and a secondary anti-IgG HRP-conjugated antibody (goat anti-rabbit IgG [H+L]-HRP conjugate; Bio-Rad) were used to detect BspK. Blots were visualized using a ChemiDoc MP Imager. The whole MBP-BspK-His protein was used for immunization during anti-BspK antibody production. The generated antibodies have a preference for binding the BspK part of the fusion protein; however, a fraction of the antibodies are also directed against the MBP domain.

### Activity of BspK autoproteolytic fragments.

BspK was preincubated at −80 or 37°C overnight, followed by a subsequent incubation with IgG1 at 37°C overnight (20 mM Tris, pH 6.8, containing 2.7 mM CaCl_2_). Proteins were separated by SDS-PAGE under reducing conditions, followed by Western blotting. Primary antibodies directed to BspK (anti-BspK/anti-MBP polyclonal, rabbit antibody) and secondary anti-IgG HRP-conjugated antibodies (goat anti-rabbit IgG [H+L]-HRP conjugate; Bio-Rad) were used to detect BspK. Blots were visualized using a ChemiDoc MP Imager.

### BspK immunoglobulin substrates.

The extent of BspK activity on immunoglobulins was investigated by including all human immunoglobulin isotypes (IgG, IgA, IgE, IgD, and IgM), all hIgG subclasses (IgG1 to IgG4), and a wide array of different animal IgG antibodies in a standardized activity assay (20 mM Tris, pH 6.8, containing 2.7 mM CaCl_2_). To evaluate the effect of prolonged incubations, the sample sets were incubated for 2 and 24 h. The variation in the IgG1 to IgG4 hinge regions was visualized by sequence alignment (ClustalW). Accession numbers for the human IgG constant regions applied in the alignment are as follows; IGHG1, P01857.1; IGHG2, P01859.2; IGHG3, P01860.2; and IGHG4, P01861.1.

### Identification of preferential sites of hydrolytic activity of BspK by HPLC.

BspK was incubated with a diverse set of peptides (Table 3) overnight at 37°C at an enzyme/peptide ratio of 1:20 in 20 mM Tris-HCl, pH 6.8, containing 100 mM NaCl and 2.7 mM CaCl_2_. Trypsin and LysC digestions were performed overnight at room temperature at enzyme/peptide ratios of 1:40 in 0.1 M Tris, pH 8.0, and 1:20 in 50 mM ammonium acetate, pH 8.5, respectively. Digestion of peptides was analyzed using reverse-phase high-performance liquid chromatography (HPLC) on an Agilent 1260 Infinity instrument with an Agilent Poroshell 120 EC-C_18_ column (2.7-μm particle size; 2.1 by 50 mm) using a linear gradient of acetonitrile-trifluoroacetic acid (TFA). Absorbance at 215 nm was recorded.

**TABLE 3:** BspK hydrolyzes its substrates C-terminally of lysine residues^*a*^

Peptide	Sequence	Activity
MOG	MEVGWYRSPFSRVVHLYRNG**K**	No
H2686	YIYGSF**K**	No
H4062	**KK**LVFFA	Yes
Apo-myoglobulin	MGLSDGEWQQVLNVWG**K**VEADIAGHGQEVLIRLFTGHPETLE**K**FD**K**F**K**HL**K**TEAEM**K**ASEDL**KK**HGTVVLTALGGIL**KKK**GHHEAEL**K**PLAQSHAT**K**HKIPI**K**YLEFISDAIIHVLHS**K**HPGDFGADAQGAMT**K**ALELFRNDIAA**K**Y**K**ELGFQG	Yes

a Reverse-phase HPLC screening was done with short peptides and apo-myoglobulin to identify BspK preferential sites of hydrolytic activity. Lysines are indicated in bold.

### Identification of preferential sites of hydrolytic activity by mass spectrometry.

Proteins were separated by SDS-PAGE. Excised protein bands were reduced (with dithiothreitol [DTT]; Sigma) and alkylated (with iodoacetamide; Sigma) and subjected to either combined LysC (lysyl endopeptidase; Wako)-trypsin (Promega) digestion or to digestion with chymotrypsin (Promega) or trypsin alone. Extracted peptides were analyzed by LC-MS/MS (Ultimate 3000 nano-HPLC system coupled to a Q-Exactive Plus mass spectrometer; Thermo Scientific). Peptides were separated on a C_18_ column (12 cm by 75 μm, 3-μm beads; Nikkyo Technologies) at 300 nl/min with a gradient increasing from 5% buffer B–95% buffer A to 50% buffer B–50% buffer A in 81 min (buffer A, 0.1% formic acid; buffer B, 80% acetonitrile, 0.1% formic acid) and analyzed in a data-dependent (DDA) manner. MS/MS data were extracted and queried against a protein database containing the target protein sequences concatenated with E. coli as a background proteome and known common contaminants (47) using Proteome Discoverer 1.4 (Thermo Scientific) and MASCOT 2.5.1 (Matrix Science). Values of 10 ppm and 20 mDa were used as mass accuracy for precursors and fragment ions, respectively. Data from the three types of digestions were merged and searched without enzymatic constraints. Matched peptides were filtered using a 1% false discovery rate calculated by Percolator (48) and, in addition, requiring that a peptide was matched as rank 1 and that precursor mass accuracy was better than 5 ppm. The area of matched peptides was used to estimate the sites of cleavage.

## Supplementary Material

Supplemental material
