# Supplementary material for: BspK, a Serine Protease from the Predatory Bacterium Bdellovibrio bacteriovorus with Utility for Analysis of Therapeutic Antibodies
Source: Appl Environ Microbiol. 2017 Feb 1;83(4):e03037-16. doi: 10.1128/AEM.03037-16 (PMC5288813; doi:10.1128/AEM.03037-16)
Supplement: Supplemental material [file supp_83_4_e03037-16__index.html]

Supplemental material 

# BspK, a Serine Protease from the Predatory Bacterium Bdellovibrio bacteriovorus with Utility for Analysis of Therapeutic Antibodies

## Supplemental material

- Supplemental file 1 -

  XtalPred prediction of BspK suggests a β-strand structured protein (Fig. S1), BspK autoproteolytic activity is temperature dependent (Fig. S2), autoproteolysis of native BspK (Fig. S3), activity of BspK autoproteolytic products (Fig. S4), BspK hydrolysis of the H4062 peptide, resembling the activity of LysC (Fig. S5), BspK hydrolysis of apo-myoglobulin, resembling the activity of trypsin and LysC (Fig. S6), apparent distant relationship of BspK to most proteases (Fig. S7), conserved nature of BspK among predatory bacteria (Fig. S8), and ClustalW alignment of closely related BspK proteins (Fig. S9).

  PDF, 1.2M
