# Supplementary material for: BspK, a Serine Protease from the Predatory Bacterium Bdellovibrio bacteriovorus with Utility for Analysis of Therapeutic Antibodies
Source: Appl Environ Microbiol. 2017 Feb 1;83(4):e03037-16. doi: 10.1128/AEM.03037-16 (PMC5288813; doi:10.1128/AEM.03037-16)
Supplement: Supplemental material [file AEM.03037-16_zam999117660s1.pdf]

```
1...*...10....*...20....*...30....*...40....*...50....*...60....*...70....*...80....*...90....*..100
MNKSFLTTMGVAVLATMTVGFVNVGPRVIYGEDNRVDIYEVSRADYRELADSTVALIPKRNIAIEANGEIKIRGGKFGTERRLCSDEPFFDQVVAANCSG

....*..110....*..120....*..130....*..140....*..150....*..160....*..170....*..180....*..190....*..200
SLVGEDLIATAGHCVSNSDCSSYNFVFGFTMQNSNTLRTLSSDDVYSCKEIVAREYTRNQDYALVRLDRAVKNRLPLKMQKTPAQPGEEIFVIGHPSGLP

....*..210....*..220....*..230....*..240....*..250....*..260....*..270....*..280....*...
SKFADGAAVRTQVGAYFQANLDTYGGNSGSAVFNSRTKEIVGILVRGSNDFSYDRERQCTASAVCPDNGCRGEDVSNISDIINAVNKL
```

Fig. S1. XtalPred prediction of BspK suggests a β-strand structured protein. The BspK amino acid sequence from *Bdellovibrio bacteriovorus* strain HD100 was subjected to analysis through XtalPred. Amino acids marked with red (alpha helix), blue (β-strand), or underlined (disordered regions) details the proposed secondary structure of the protein.

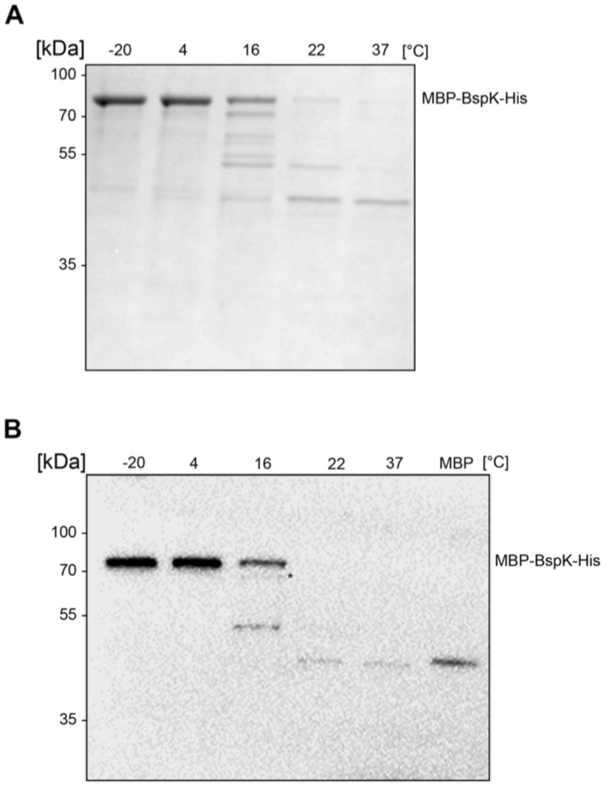

Fig. S2. BspK autoproteolytic activity is temperature dependent. A) SDS-PAGE showing that BspK autoproteolysis is reduced at lower temperatures B) Anti-BspK/-MBP western blot. Intact recombinant BspK (MBP-BspK-His) has a molecular weight of ~74 kDa; MBP has a molecular weight of 42.5 kDa. Asterisk used to highlight the K_{210} hydrolysis product. All samples were incubated for 16 h in 20 mM Tris pH 6.8 supplemented with 2.7 mM $CaCl_2$.

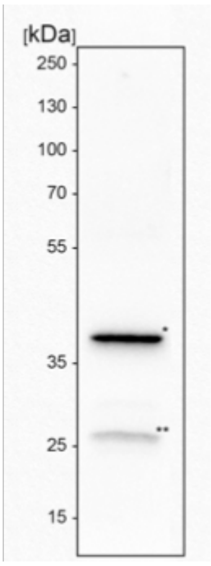

Fig. S3. Autoproteolysis of native BspK. Anti-BspK/-MBP western blot showing autoproteolysis of native BspK. Intact native BspK has a theoretical molecular weight of ~32 kDa. Bands marked with an asterisks indicate intact BspK* and a BspK autoproteolytic fragment**.

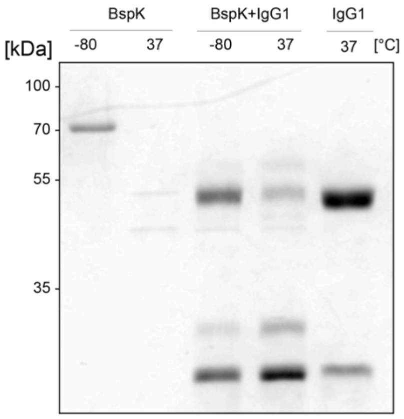

Fig. S4. Activity of BspK autoproteolytic products. A) SDS-PAGE showing that the autoproteolytic products of recombinant BspK are enzymatically active. BspK was pre-incubated over night at -80°C or 37°C (lane 1, 2), before addition of IgG1 and continued incubation over night at 37°C (lane 3, 4). Non-hydrolyzed IgG1 was used as a control (lane 5). Intact recombinant BspK (MBP-BspK-His) has a molecular weight of ~74 kDa; MBP has a molecular weight of 42.5 kDa.

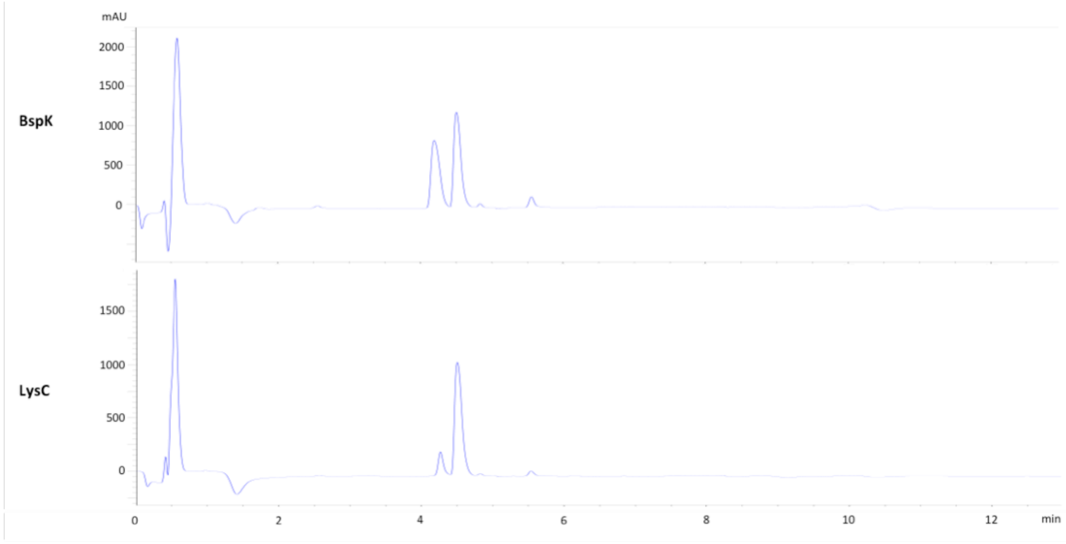

Fig. S5. BspK hydrolysis of the H4062 peptide resembles the activity of LysC. The small H4062 peptide KKLVFFA was incubated with BspK (enzyme: peptide ratio 1:20), and LysC in their respective buffers (overnight, 37°C). The BspK cleavage profile was established by comparison to LysC hydrolysis of the peptide. Digested peptides were analyzed using reverse phase HPLC.

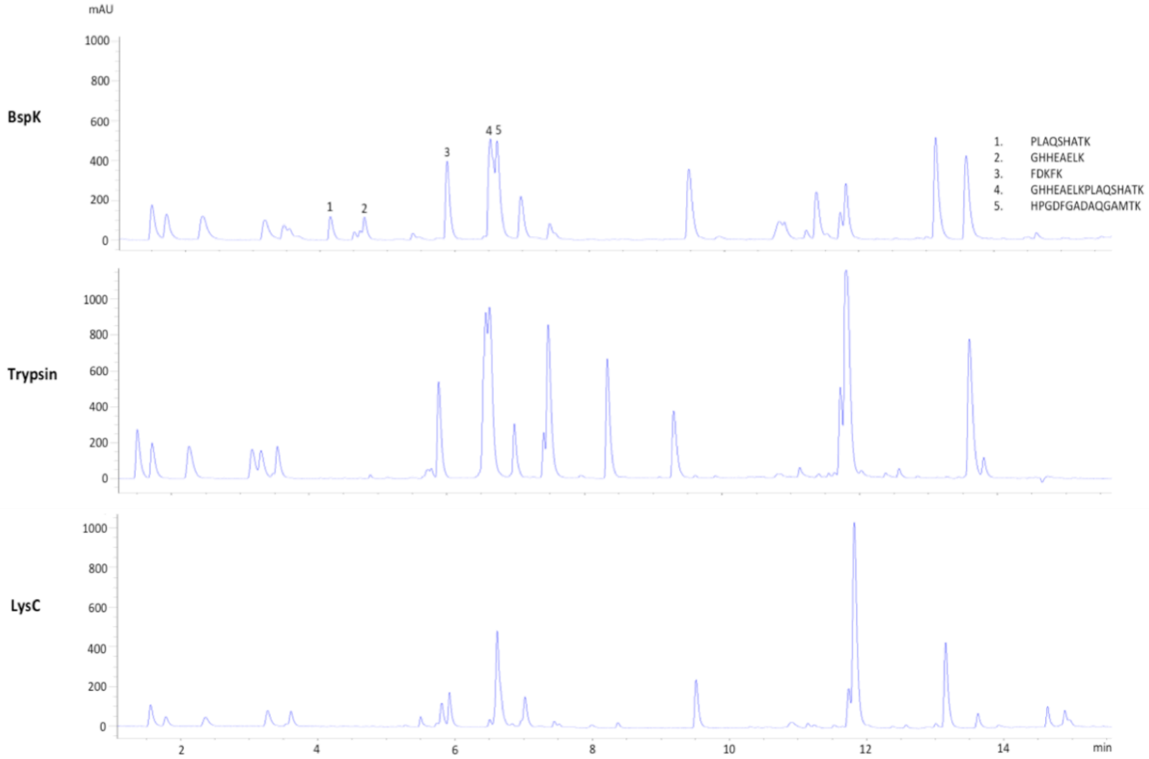

Fig. S6. BspK hydrolysis of apo-myoglobulin resembles the activity of trypsin and LysC. Apo-myoglobulin (Table 3) was incubated with BspK (enzyme: peptide ratio 1:20), trypsin, and LysC in their respective buffers (overnight, 37°C). Digested peptides were analyzed using reverse phase HPLC and mass spectrometry.

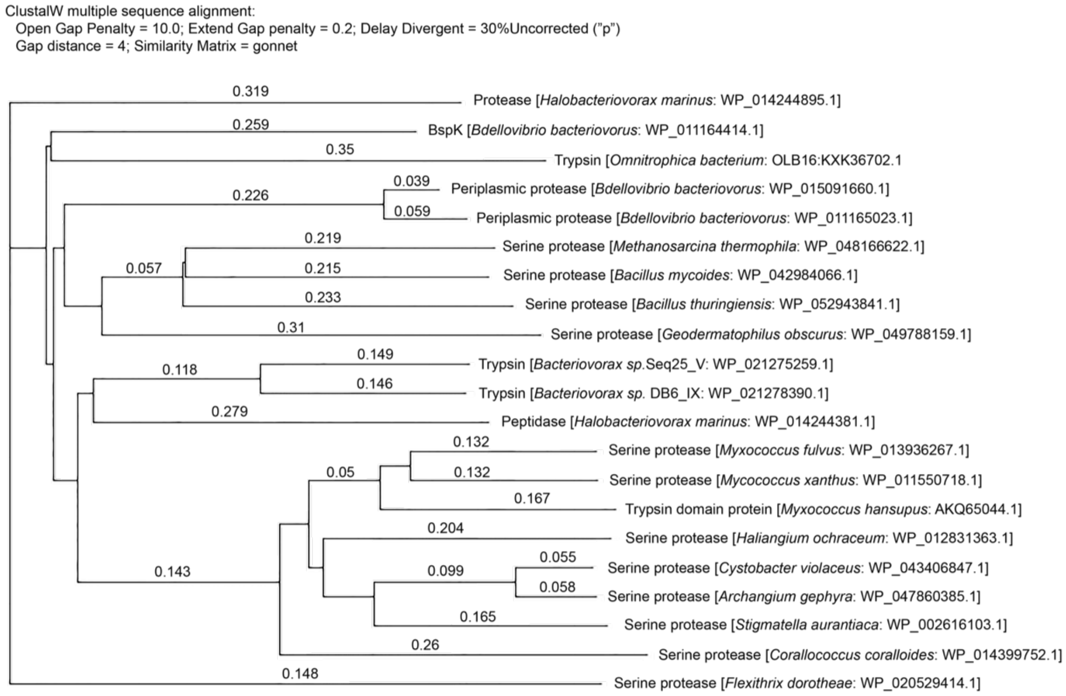

Fig. S7. BspK appears to be distantly related to most proteases. Similarity tree based on ClustalW multiple sequence alignment of BspK and related bacterial proteases.

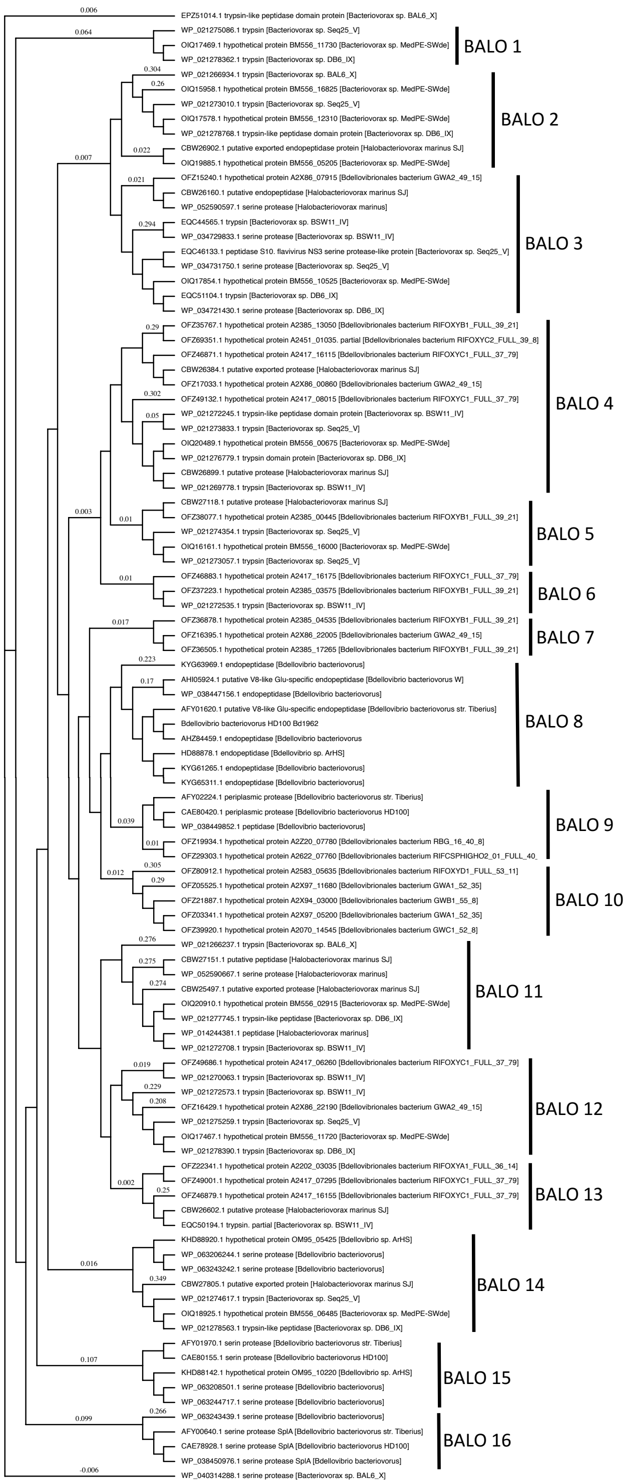

Fig. S8. Protein Blast of BspK in the order Bdellovibrionales indicates the conserved nature of BspK amongst predatory bacteria. A BLAST against BspK was conducted, limited to the order Bdellovibrionales. The 100 top hits were used to generate a guide tree.

```
BALO_8A         1 MTKKSILTSLLVPTLASLCVGFVNVTPKVIYGDDNRADVYEIQDPVVREVANSTVAIIHK  60
BALO_8B         1 MN-KSFLTTMGVAVLATMTVGFVNVGPRVIYGEDNRVDIYEVSRADYRELADSTVALIPK  59
BALO_8C         1 MKRNSLVASISVAVAATFSVGFVNITPKVIYGEDNRVDVYQVQRADIREIADSTVALIPS  60
BALO_8D         1 MKRNSFVATMSVALAATFSVGFVNITPKVIYGDDNRVDVYQVTRSDIREIADSTVALIPT  60
                  *     .... *   *. .*****. *.****.*** *.*..     **.* ****.*

BALO_8A        61 KFLKPDGQGRYIFTSTRFGRSQDMCSEEPYYNQPDAAVCSGSLVGEDLIATAGHCVNHST 120
BALO_8B        60 RNIAIEANGEIKIRGGKFGTERRLCSDEPFFDQVVAANCSGSLVGEDLIATAGHCVSNSD 119
BALO_8C        61 RAVVRDAQGNFKINTTTYGHELDLCKSEPFFDQPTAANCSGSLVGDDLIATAGHCISTKD 120
BALO_8D        61 RDIQNQGNGLIKILASSFGTSMNLCSSEPFFDQPAAANCSGSLVGEDLIATAGHCVSNAD 120
                  . .    .*        .*    .*  **.. *  ** *******.*********.

BALO_8A       121 CKNYYYVFGYQMTDSKTAPESFAAEEIYTCKGIVAREYTNDEDFALLRLNRKVPNHRPLT 180
BALO_8B       120 CSSYNFVFGFTMQNSNTLR-TLSSDDVYSCKEIVAREYTRNQDYALVRLDRAVKNRLPLK 178
BALO_8C       121 CGTYSFVFGFRMLDAKTAPQTISADDVYTCKEIVAREYTSAQDYALVRLDRPVRGHRVLA 180
BALO_8D       121 CSRYAFVFGYKMTDAKTAPDTVSESDVYSCKEIVAREYTGAQDYALVRLDRPVRGHRVLT 180
                  *  * .***. *  . *   . .  ..*.** *******  .*.**.** * * ..  *

BALO_8A       181 LQRAPAQEGDEVYVVGHPSGLPTKVAGGATVREQEGRYFSANLDSYGGNSGSAVLNARTH 240
BALO_8B       179 MQKTPAQPGEEIFVIGHPSGLPSKFADGAAVRTQVGAYFQANLDTYGGNSGSAVFNSRTK 238
BALO_8C       181 IQSTPVQPGDDIYVVGHPSGLPTKIADGAKVRSQKNGYFVTNLDTYGGNSGSAVFNARTN 240
BALO_8D       181 LAQTPARPGDAIYVVGHPSGLPTKVADGAQVRAQQGAFFTSNLDTYGGNSGSAVFNARTN 240
                  .  .* . *. ..*.*******.* * ** ** * . .* .***.********* *.**

BALO_8A       241 EVVGILVRGSQDFKYDQARMCYRSNVCDNDGCGGEDVTNISYIADALNQTTR 292
BALO_8B       239 EIVGILVRGSNDFSYDRERQCTASAVCPDNGCRGEDVSNISDIINAVNKL   288
BALO_8C       241 EIVGILVRGAQDFAYDRVNQCTVSNRCTDDGCRGEDVTNISFISQALKQ    289
BALO_8D       241 EVVGILVRGAQDFTYDRTKQCTVSNKCTEAGCRGEDVTNISYIVNALKK    289
                  *.*******..** **.   *  *  *   ** ****.*** *  *. .
```

Fig. S9. ClustalW alignment of closely related BspK proteins indicate a variability in autoproteolytic regions. Four different subgroups of the BspK BALO_8 cluster were aligned using the ClustalW alignment, with 8C representing the BspK type used in this study. K_{28} and K_{210} are highlighted with blue, demonstrating a variability in the presence of a lysine both at position 28 (3/4) and at position 210 (1/4).
